# Diversity of Arbuscular Mycorrhizal fungi under different agroforestry practices in the drylands of Southern Ethiopia

**DOI:** 10.1186/s12870-023-04645-6

**Published:** 2023-12-09

**Authors:** Nebiyou Masebo, Emiru Birhane, Serekebirhan Takele, Zerihun Belay, Juan J. Lucena, Araceli Pérez-Sanz, Agena Anjulo

**Affiliations:** 1 Department of Natural Resource Management, Wolaita Sodo University, Wolaita Sodo, P.O. Box 128, Ethiopia; 2 Department of Land Resource Management and Environmental Protection, Mekelle University, P.O. Box 231, Tigray, Ethiopia; 3 Department of Biology, Arba Minch University, Arba Minch, P.O. Box 138, Arbaminch, Ethiopia; 4 Department of Applied Biology, Adama Science and Technology University, P.O. Box 231, Adama, Ethiopia; 5 Department of Agricultural Chemistry and Food Science, Autonomous University of Madrid, Madrid, 28049 Spain; 6 Department of Agricultural Chemistry and Food Science, Universidad Autónoma de Madrid, Madrid, 28049 Spain; 7 Environment and Forest Research Institute, Addis Ababa, P.O. Box 231, Ethiopia; 8 Institute of Climate and Society, Mekelle University, P. O. Box 231, Mekelle, Ethiopia; 9 Faculty of Environmental Sciences and Natural Resource Management, Norwegian University of Life Sciences (NMBU), Ås, Norway

**Keywords:** Agroforestry practices, Root colonization, Species diversity, Spore density, Soil Properties

## Abstract

The conversion of an agroforestry based agricultural system to a monocropping farming system influences the distribution and composition of arbuscular mycorrhizal fungi (AMF). The aim of this paper was to analyze AMF species diversity, spore density, and root colonization across different agroforestry practices (AFP) in southern Ethiopia. Soil and root samples were collected from homegarden, cropland, woodlot, and trees on soil and water conservation-based AFP. AMF spores were extracted from the soil and species diversity was evaluated using morphological analysis and root colonization from root samples. The AMF spore density, root colonization and composition were significantly different among the AFP (*P* < 0.05). In this study, 43 AMF morphotypes belonging to eleven genera were found, dominated by *Acaulospora* (32.56%), followed by *Claroideoglomus* (18.60%). Home gardens had the highest spore density (7641.5 spore100 g^− 1^ dry soil) and the lowest was recorded in croplands (683.6 spore100 g^− 1^ dry soil). Woodlot had the highest root colonization (54.75%), followed by homegarden (48.25%). The highest isolation frequency (63.63%) was recorded for *Acaulospora scrobiculata*. The distribution of AMF species and diversity were significantly related to soil total nitrogen and organic carbon. The homegarden and woodlot AFP were suitable for soil AMF reserve and conservation.

**Supplementary Information:**

The online version contains supplementary material available at 10.1186/s12870-023-04645-6.

## Article highlights

The homegarden and woodlot agroforestry practices are the best alternative mechanisms for conservation of soil AMF biodiversity.

The agroforestry practices that are known with high surface vegetation cover results in conservation of soil AMF.

The agricultural practices such as scattered trees on farm-based systems with intensive tillage impacts soil AMF communities.

**Supplementary Information:**

The online version contains supplementary material available at 10.1186/s12870-023-04645-6.

## Introduction

In tropical regions, habitat loss and fragmentation are the major threats to biodiversity, and much of this is driven by agriculture [1]. In southern Ethiopia, the land degradation has been increasing due to high population density and fragmented farmland as well as continuous farming [2]. Forests have been converted mainly into agroforestry systems (AFS) and further into agricultural systems with gradual replacement of an age-old diverse coffee-enset (*Ensete ventricosum (*Welw.) based AFS with a mono-cropping system [3, 4]. In this area, inclining towards monocropping has been causing an intensive land use and forest clearing for cultivation even in areas that are not suitable for agriculture [5–7]. On top of this, conversion of tree-based systems into monocropping influences both the aboveground and belowground community structure. Arbuscular mycorrhizal fungi (AMF) are one of the belowground systems that significantly affected by land use change. The agroforestry approach could be one of the viable options for promotion of AMF communities, however, there are different challenges that could hinder the implementation of AFP including the lack of knowledge and limited technical inputs to farmers [8], the lack of cash flow and financial access [9] to local communities. AFP is labour intensive activity that incurs high cost during implementation [10] and the poor skills among extension agents [11] hinder the expansion of AF practices. Intensive management activities that facilitate monocropping negatively affects the diversity and distribution of AMF.

Arbuscular mycorrhizas fungi (AMF) are the most widespread land plant–fungus mutualisms that colonizes at least 72% of flowering plant species [12]. Some vascular plants such as *Deschampsia antarctica* Desv (grass species) and *Colobanthus quitensis* (kunth) are colonized by few AMF species [13]. The AMF are known to be important for the stability and productivity of ecosystems [14]. The dependency of ecosystems on microbial organisms is due to the effects of microbial abundance and diversity, which will be of significance to the functioning of these systems [15]. Healthy ecosystems are more resistant and resilient to mitigate climate change and other impacts [16]. AMF improves plant growth parameters [17] and the uptake of several major nutrients in normal and stressed conditions [18]. Moreover, AMF are crucial for the protection of their hosts against abiotic [19, 20] and biotic factors [21]. However, there are factors that could influence the AMF diversity and community composition. Types of host plant species have a strong and significant effect on AMF diversity and distribution [22], higher AMF spore abundance was registered under legume tree based AFP, than monoculture based agriculture [23]. Changing the vegetation cover from tree-based intercrops to mono-cropping system can reduce AMF fungal richness [24]. The edaphic factors including soil type and property could decide the AMF richness. Soil type strongly affect AMF composition and the occurrence of species [25]. The differences in soil property have been determining AMF community composition [26], like soil pH [23], soil organic carbon [24], soil nitrogen [25], and land use intensity [26]. Land use management practices and climatic variables are crucial factors that can decide the AMF composition. High richness of AMF were enumerated under less disturbed land use systems and [27] with significantly greater amount of AMF in a tree-based AF system compared to an adjacent mono-cropping system. Climatic variables such as rainfall, relative air humidity, and precipitation influences AMF community composition, sporulation [28], spore density and richness [29]. Both natural and anthropogenic factors contribute to the diversity and distribution of AMF in an agroforestry land use system.

The structure of AMF communities has been studied across many ecosystems. For instance, higher spore densities and species richness were reported in natural ecosystems [26], grassland ecosystems [30], agroecosystems [31], and wetland ecosystems [32] compared to agricultural land. Changes in AMF communities under various land covers were the result of land use change in different areas of the tropics [33]. Likewise, the impacts of agricultural intensification, land use conversion, and the loss of different plant species on microbial abundance and diversity were reported [34]. Intensive land use change for several decades has caused a reduction in abundance and diversity of AMF under monocropping-based agriculture in Ethiopia [35]. Intensive agricultural activities that can bring land use change can destroy the large biodiversity and plant community structure. Plant community structure affects the diversity, community composition and species richness of AMF [36]. AMF species richness and diversity are determined by plant diversity [33]. AMF diversity and abundance in soil are influenced by vegetation cover [37]; the lower AMF species richness was reported from arable fields as compared to natural ecosystems and perennial tree based systems [38]. Moreover, soil physicochemical properties and depth variation can affect the community structure of AMF [26, 33].

Agroforestry (AF) is a sustainable land use practice geared to harmonize ecosystem productivity and conservation [39]. The AF is also hypothesized to harbor a relatively high AMF species richness and abundance due to the increased density of host plants compared to monocultures [40]. The evidence indicated that AF had a positive influence on the composition of the AMF community compared to conventional and native forest land [41]. Similarly, higher numbers of spores from AFS were reported than monoculture coffee cultivation [42]. AF has been considered key to supporting a more abundant and diverse AMF community than conventionally managed systems [38, 43, 44]. Home garden, parkland, alley cropping, trees on soil and water conservation structures, woodlot, border planting, windbreaks, and shelter belt agroforestry practices are AF land management practices that could promote the abundance and diversity of AMF.

AMF diversity and distribution have been studied in different land uses, ecosystems and agroecologies in Ethiopia. AMF root colonization and spore density were documented in the dry afromontane forest, fragmented church natural forest and different land use types in northern Ethiopia [45–49]. AFPs have been widely practiced in south Ethiopia for enhancing crop production, water and energy use efficiency, and soil health. Abundance and composition of AMF under different land use types using trap culture and field [50], and AMF richness under native forest versus agroforestry has been analyzed [37]. AMF community under savannah ecosystem of Nachi Sar national Park reported higher AMF richness and diversity from un-encroached plots [51]. Moreover, the AMF community composition, richness, and diversity on enset (*Ensete ventricosum* (Welw.) Cheesman) had higher number of *Acaulospora* species from less manured farm compared to manured field [52]. Change from an AFP into monocropping is affecting AMF community structure. The aim of this paper was to investigate AMF diversity and structure between the different AFP in the drylands of southern Ethiopia. The soils and root samples were collected from two depths to analyze (i) whether AMF composition, richness, diversity, dominance and evenness differs between AFP, (ii) whether AMF community structure varies between soil depths, (iii) whether AMF spore density varies along the AFP and soil depth, and root colonization between AFP, (iv) analyze the relationship between soil properties, AMF composition, spore density and root colonization, and (v) analyze the interaction effect of soil depth and AFP on AMF composition and spore density in the drylands of southern Ethiopia. We hypothesize that AMF species diversity, root colonization and spore density between AFP differs because of the difference in plant diversity, host plant identity, and soil management practices. The findings of the paper will contribute to properly understand management of soil health under a monocropping system and an agroforestry system.

## Methods

### Study area

**Fig. 1:**
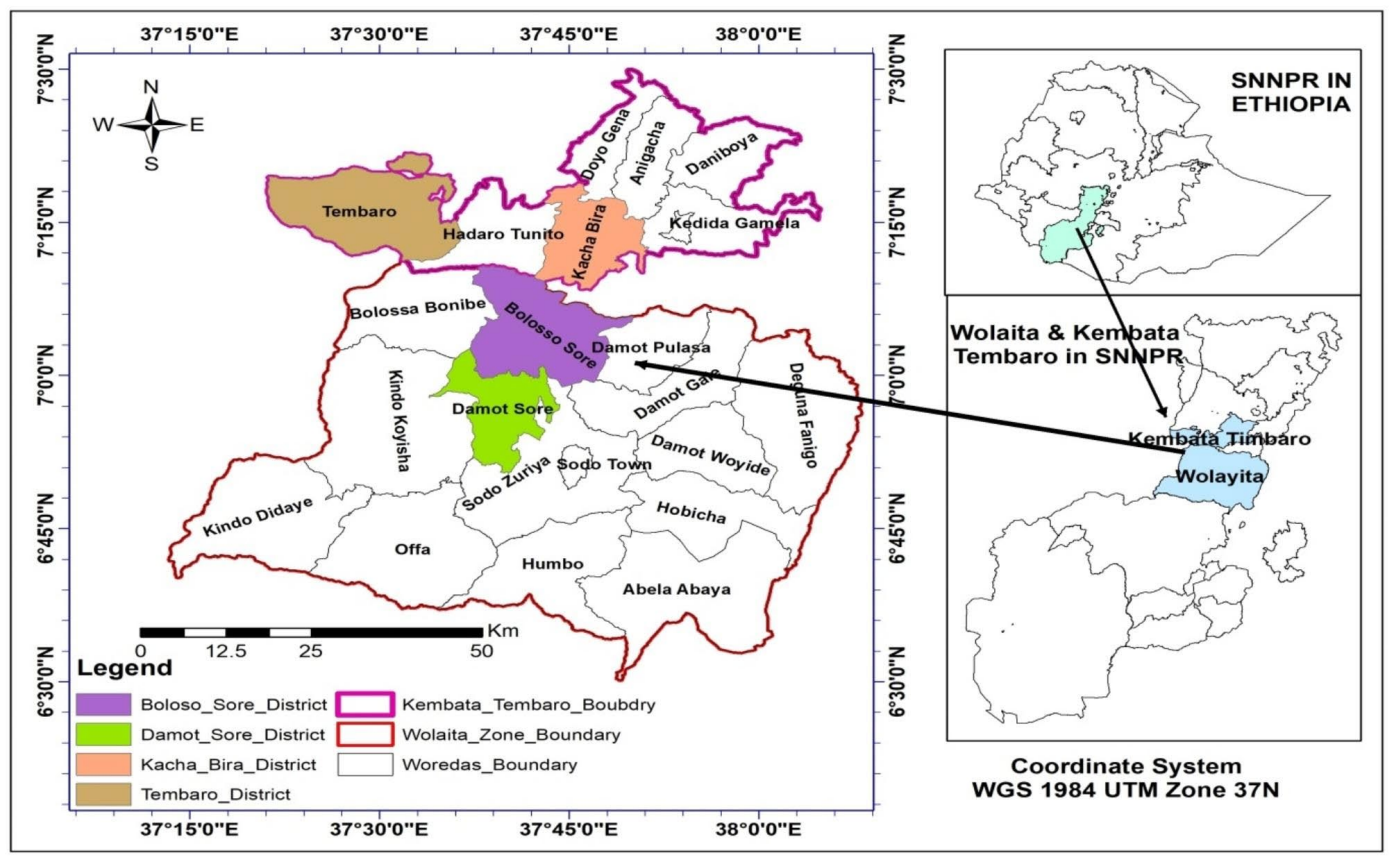
Map of the study area showing the location of the study districts in Wolaita and Kembata Tembaro zones in south nations and nationalities people’s regional state in Ethiopia (Figure produced using ArcGIS 10.8.1 software by the authors with data source from the central statistics authority (CSA, 2007) in Ethiopia)

The study was conducted in southern Ethiopia which is located in southern and southwestern part of the country (5° 50’ 26’’– 6° 12’ 48’’ N, 38° 03’ 02’’–38° 18’ 59’’ E) [7]. AFP are predominant practices in the region. The practices are categorized under the high potential perennial zones. Enset and coffee are grown in an intimate association with other crops, trees and livestock in multistorey homegarden AFP [4] that are widely practiced in most of the administrative zones of the region. Wolaita, and Kembata Tembaro zones were selected to conduct the study **(**Fig. 1) which represents diverse types of AFP (Table 1). Wolaita zone is located at 037°35ʹ–037°58 ʹE and 06°57ʹ–07°04 ʹN. The zone has an altitude between 650 and 2900 m above sea level. The annual rainfall is between 700 and 1480 mm. Kembata Tembaro zones is found 037^0^34‘-38^0^ 07‘E and 07^0^10‘-7^0^61‘N with an altitude of 700–3028 m and receives an annual rainfall between 900 and 1400 mm.

The zones have a bimodal rainfall with small rains from March to May and heavy rains during July and August. The mean annual temperature is 20.1 °C [53]. The dominant soils are Nitosols [54], with moderately to strongly acidic pH [55]. The main food crops are maize (*Zea mays* L.), beans (*Phaseolus vulgaris* L.), sweet potatoes [*Ipomoea batatas* (L.), and enset (*Ensete ventricosum)*. Teff (*Eragrostis tef*), coffee (*Coffea arabica*), and ginger (*Zingiber officinale* Ros.) are among the cash crops cultivated. Cattle, sheep, poultry, and donkey are the main livestock types.

**Table 1:** Characteristics of the Agroforestry practices (AFP) in the drylands of southern Ethiopia

No.	Agroforestry types	Agricultural practices	Vegetation composition	Management activities
1	Homegarden based agroforestry practices (HAFP).	No use of fertilizer, no grazing, selective cutting.	*E. ventricosum*, and trees species such as *C. arabica, P. americana, M. indica, G. robusta, C. africana*, and *F. vasta* covered > 75% of the land area and is dominated with dense number of different vegetables and herbs.	Pruning, thinning, weeding, composting, and application of organic manures.
2	Cropland based agroforestry practices (ClAFP).	High use of fertilizer, selective cutting, grazing after crop harvest.	Sparse types of trees species like *P. americana*, *M. indica, C. africana, F. vasta and C. macrostachyus*.	Pruning, grass mulch, application of inorganic fertilizers and weeding
**3**	Woodlot based agroforestry practices (WlAFP).	No of use fertilizer, grazing selective cutting.	Dense number of Eucalyptus species and tress like acacia species, *C. macrostachyus, J. procera* grows.	Pruning, thinning, coppicing, pollarding, and weeding.
4	Trees on soil and water conservation-based agroforestry practices (TSWAFP).	High use of fertilizer and grazing after crop harvest.	Sparse types of trees species such as *C. macrostachyus, musa, V. auriculifera* and herbs like grass species grow on physical SWC structures.	Pruning, grass mulch, application of inorganic fertilizers and weeding.

### Sampling design

A multistage sampling design was used to select the zones, districts, and villages from the southern region of Ethiopia. The southern region was selected among the different states based on its potential AFPs. The four districts and villages were selected using stratified sampling techniques based on slope, type of AFP, altitude, soil, and agroecology. The AFP at village level was selected randomly among the homogenous units of the AFPs. The soil and root samples were collected from homegarden based agroforestry practice (HAFP), cropland-based agroforestry practice (ClAFP), woodlot-based agroforestry practice (WlAFP), and trees on soil and water conservation-based agroforestry practice (TSWAFP). Soil and root samples were collected using a 10 m x10 m plot size for WlAFP [56], 25 m x 25 m plot size for HAFP [57], 40 m x 40 m plot size for ClAFP and TSWAFP [58]. Data collection was done using 128 samples from four AFP in four districts, two villages composed of two farms each at two depth levels of 0–30 cm and 30–60 cm. Soil physicochemical parameters were collected from randomly placed 1 m x 1 m square plots distributed at the corners and center of the main plots following an “X” pattern. The rhizosphere soil was collected from all the woody species found in each plot and the dominant woody species from each plot were replicated three times.

### Soil and root sample collection

The soil samples were collected at 0–30 and 30–60 cm soil depth levels using an auger. Gravel materials and dead plant materials were excluded from the collected soil samples. One kilogram of each of the 128 composite soil samples was packed in plastic bags and tagged separately in terms of its replication, and depth categories. The collected and cleaned soil samples were air dried and transported to southern regional soil laboratory in Hawassa, Ethiopia, for soil analysis.

The soil particle size was determined using the hydrometer method [59]. The organic carbon was determined by the Walkley-Black procedure [60]. Total nitrogen (TN) was determined by the Kjeldahl method [61]. The soil available phosphorus was measured according to the method described by Olsen [62] and soil pH was measured by deionized water in a 1:2.5 soil: water suspension [63].

The soil samples for AMF spore density analysis were collected from four corners of the woody species at two depth levels (upper = 0–30 cm and bottom = 30–60 cm). The selected dominant woody species in each plot were replicated three times [43]. Soil samples were collected from thirteen dominant woody species in the HAFP and ClAFP while eight and sixteen dominant woody species were selected from TSWAFP and WlAFP, respectively and replicated three times. AMF spore density (SD) was enumerated using three hundred soil samples using seventy-eight samples each from HAFP and ClAFP (13 × 3 × 2), forty-eight sample from TSWAFP (8 × 3 × 2) and ninety-six samples from WlAFP (16 × 3 × 2). Root colonization analysis was done using 150 root samples collected from the selected dominant woody species in each plot from one depth level.

We analyzed AMF root colonization using fifty woody species distributed among four AFP types (13 each from HAFP and ClAFP, eight from TSWAFP and sixteen from WlAFP). The root samples of all selected dominant woody species were collected by excavating soil starting from the plant’s trunk base in four directions of the plant and working outwards to get live fine roots within a 3–5 m radius [64]. A total of 150 woody species root samples measured 5 g with a diameter < 2 mm composite sample from all four sampling points was collected for laboratory analysis. The collected root samples were washed with tap water to remove any soil particles and put into tightly sealed plastic jar, which was filled with 97% ethanol to preserve the roots. Root samples were stored at 4 °C room temperature until they were ready for further laboratory analysis in Adama Science and Technology University microbiology laboratory.

### Root staining and quantification of AMF root colonization

Roots were washed carefully with tap water and cut into segments of about one cm long and put in a test tube (15 ml) having 10% (w/v) KOH and heated at 90 °C in a water bath for one hour to effect clearing. The roots were washed to remove the KOH and treated with 10% HCl (v/v) for 15 min at room temperature and finally stained in 0.05% w/v trypan blue in lactoglycerol (1:1:1 lactic acid, glycerol and water) at 90 °C for 30 min in a water bath [65]. Fungal colonization was quantified using the magnified intersection method [66] under a compound-light microscope at a magnification of x200. Thus, to examine the presence and percentage colonization of AMF structures (hyphae, arbuscule, and vesicle) root segments were mounted on microscope slides, six roots were mounted per slide from which one root segment was viewed per eyepiece.

### The occurrence, relative abundance and dominance of AMF morphospecies

**Fig. 2:**
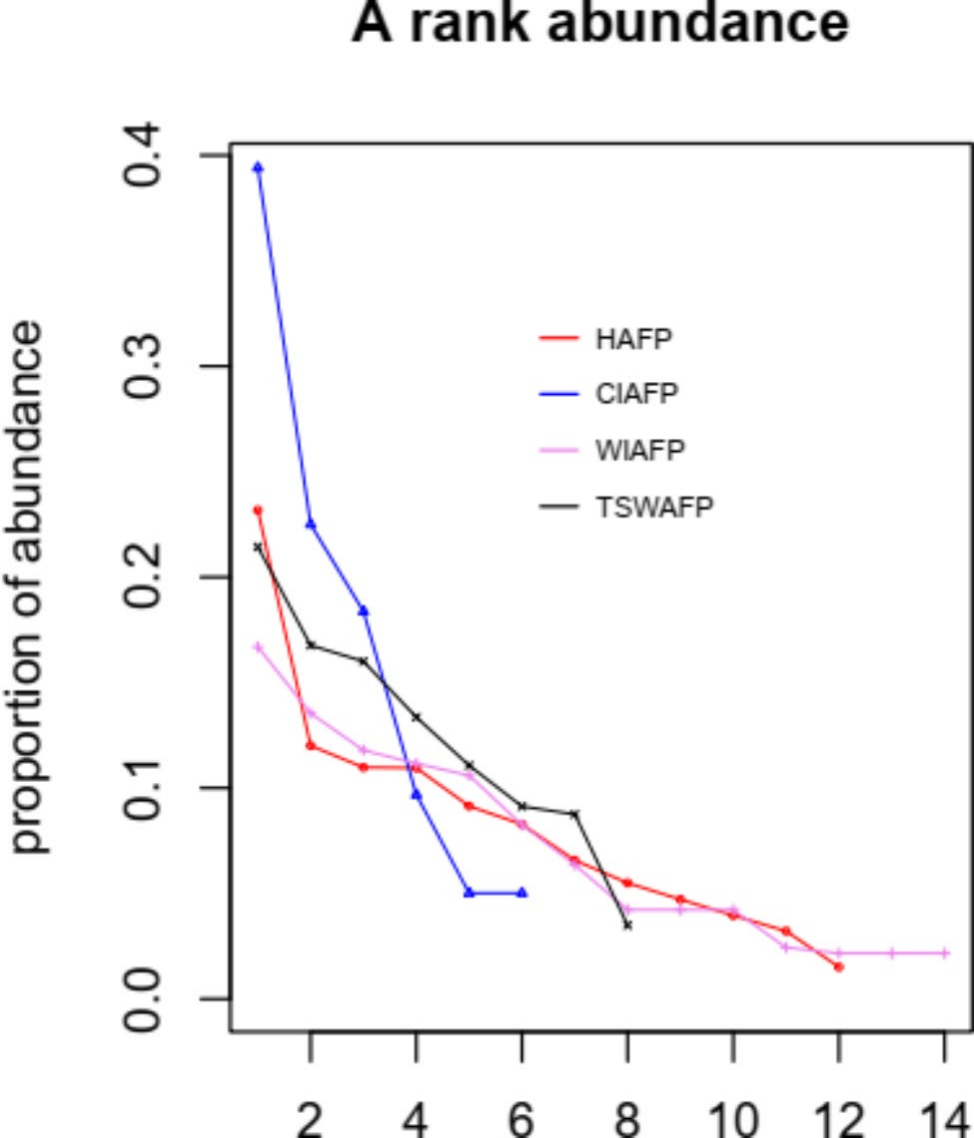
Whittaker plots illustrating the richness and abundance of the AMF spore morphospecies in the drylands of Southern Ethiopia. Numerals **x axis** refers to the list of morphospecies in Table 2. Morphospecies are plotted in sequence from highest to lowest abundance along the different agroforestry practices

The frequency of occurrence of each species was calculated based on presence or absence of the species in a sample. The frequency of the species was estimated as the number of samples in which a given species occurred as the percentage of the total number of samples. Similarly, the relative abundance of spores was calculated as the ratio of spores of a given AMF species to the total number of spores. The importance value of an AMF morphotype was estimated to evaluate the dominance of AMF species under different AFP types; important value = (isolation frequency + relative abundance)/2. The frequency > 50% was considered as a dominant, 30–50% as very common, 10–30% as common, and 10% as rare species [67]. The dominance of the AMF species was illustrated using the rank abundance Whittaker plot (Fig. 2).

### AMF spore density, richness, and diversity

Soil AMF spores from different AFP fields were isolated using the wet sieving and decanting method [68], followed by the sucrose gradient technique [65]. Hundred grams of dry soil sample was soaked in 1000 ml water and left for 5 min to settle soil particles and was decanted through five hundred µm, 350 μm, 250 μm, 180 μm, ninety µm and sixty-three µm sieve layers. The contents left in 350–63 μm sieves were collected in a test tube, suspended in water, and centrifuged at 22.4 g for five minutes, and the supernatant was decanted. The soil materials in the test tubes were re-suspended in a 50% sucrose solution and centrifuged at 22.4 g for one minute. The supernatant having the spores was poured over a 180 − 63 μm size sieve and thoroughly washed with tap water to remove the sucrose and transfer the spores to a petri dish. The AMF spores were counted under a dissecting microscope at x4 magnification. Enumeration of spore numbers per gram of dry soil was undertaken according to INVAM (http://invam.caf.wvu.edu). Thereafter, healthy looking spores with similar morphology were picked and mounted on slides in polyvinyl-lactic acid-glycerol (PVLG), and examined under the compound microscope at x400–1000 magnification [65]. The species identification and matching of morphotypes were done based on the original descriptions and identification references of species descriptions provided online by INVAM West Virginia University, USA (http://invam.caf.wvu.edu*)*, University of Agriculture in Szczecin, Poland (http://www.zor.zut.edu.pl/Glomermycota), and the Schüßler AM fungi phylogeny website (http://www.lrz.de/~schuessler/amphylo/).

AMF species richness was measured as the number of species recorded from a soil sample. AMF diversity under different AFP was evaluated according to the Shannon diversity index: H’=-∑(Piln[pi]),where Pi = ni/N, ni = number of individuals of the species i, N total number of individuals of all species and ln = natural logarithm [69]. Species evenness was calculated using the Pielou’s evenness (J) index: ,J = H’/Log(S), where H′ is the value obtained from the Shannon index (diversity), and S is the total number of AMF species present in the sample [70].

### Similarity of AMF among AFP practices

The number of AMF species shared among different AFP types were calculated using the modified Sorensen’s similarity index [71] which was used to compare more than two AMF communities between different AFP type:

$${\text{SI = }}\frac{{{\text{ab + ac + ad + bc + bd + cd - abcd}}}}{{{\text{a + b + c + d}}}}$$

, where SI is Sorenson’s similarity, a is the number of species found in AFP 1; b is the number of species found in AFP 2, c is the number of species found in AFP 3, d is the number of species found in AFP 4, ab, cd is the number of species shared by the respective two AFP types and abcd is the number of species shared among all AFP types.

### Statistical analysis

The data was checked for normality using histogram and the Shapiro-Wilk test prior to data analysis. The variation of AMF spore density, AMF species richness, diversity, and soil properties for each soil depth among the four AFP were evaluated using two-way ANOVA while the root colonization structures (hyphae, arbuscules and vesicles) were assessed using a one-way ANOVA. Tukey’s honestly significant difference (HSD) post hoc test was used for pairwise multiple mean comparisons tests between the four AFPs and between variables. The AMF species diversity, and morphotype among AFP type was determined by Shannon Wiener diversity [69] and Pielou’s [70] respectively and species richness was determined based on the number of species from the corresponding AFP type. The relationship between soil physicochemical properties and AMF spore abundance, root colonization, species richness, evenness and diversity were evaluated by multivariate multiple linear regression model. The affinity of AFP types based on morphospecies composition and soil properties were analyzed by principal component analysis (PCA). All the tests of statistical significance were decided at p < 0.05 using R statistical software version 4.2.1.

## Results

### AMF community composition

Based on morphological criteria, forty-three morphotypes belonging to eleven genera of subfamily *Glomeromycota* were characterized (Table 2). The most common genera were *Acaulospora* (32.56%) with fourteen species and *Claroideoglomus* (18.06%) with eights species followed by *Funneliformis* and *Glomus* genera each with four species. The genus *Gigaspora* had three species. *Ambispora*, *Paraglomus occultum, Rhizophagus aggregatus* and *Septoglomus* had wo species each. The genera *Scutellospora* was represented by a single species. There was one unidentified species that did not belong to the existing morphological characteristics. In this study, the identified species and morphotypes are illustrated in Figure SI 1.

**Table 2:** Number of arbuscular mycorrhizal fungi (AMF) genus morphotype characterized under different agroforestry practices in the drylands of Southern Ethiopia

AMF Genus	Number of morphotypes							
	H**AFP**		**ClAFP**		Wl**AFP**		TSW**AFP**	
	0–30 (cm)	30–60 (cm)	0–30 (cm)	30–60 (cm)	0–30 (cm)	30–60 (cm)	0–30 (cm)	30–60 (cm)
*Acaulospora* (Gerd. & Trappe)	7	0	1	1	2	1	3	0
*Ambispora* (C. Walker)	0	0	0	0	0	0	0	1
*Claroideoglomus* (C. Walker & Schuessler)	5	0	2	0	0	1	0	0
*Funneliformis* (C. Walker & Schuessler)	2	1	0	0	1	0	0	0
*Gigaspora gigantea* (Gerd. & Trappe)	1	0	0	0	1	0	1	0
*Glomus* (Tul. & C. Tul)	0	0	2	0	2	0	0	0
*Paraglomus occultum* (J.B. Morton & D. Redecker)	0	0	0	0	0	0	2	0
*Rhizophagus aggregatus* (C. Walker,)	2	0	0	0	0	0	0	0
*Scutellospora* spp	0	0	0	0	0	0	1	0
*Septoglomus constrictum* (Sieverd., G. A. Silva & Oehl)	0	0	0	0	2		0	0
Unidentified species	0	0	0	0	1		0	0

* The acronym HAFP refers to homegarden based agroforestry practices, ClAFP cropland-based agroforestry practices, WlAFP woodlot-based agroforestry practices, TSWAFP trees on soil and water conservation

### Occurrence, abundance and dominance of AMF morphospecies

**Fig. 3:**
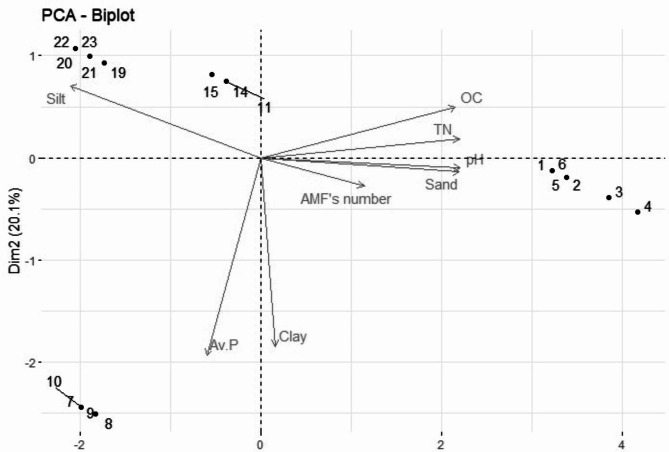
The principal component analysis (PCA) showing the affinity of AMF morphospecies composition and soil properties in relation to the agroforestry practice (AFP), where 1-6 refers to the homegarden based agroforestry practice (HAFP), 7–10 represent the crop-based agroforestry practice (ClAFP), 11–17 refers to the wood lot-based agroforestry practice (WlAFP), and 19–23 represents the trees on soil and water conservation structure-based agroforestry practice (TSWAFP). The X and Y axis is the first and second principal components, respectively

*Acaulospora scrobiculata, Claroideoglomus claroideum*, and *Glomus* spp. had the highest frequency of occurrence. *Acaulospora* genus occurred most often, followed by *Claroideoglomus*. The highest IF (63.64%) was recorded in *Acaulospora scrobiculata* followed by *Claroideoglomus claroideum* (35.23%) under HAFP and *Glomus* spp.2 (32.63%) under TSWAFP type. The RA of AMF spores was the highest for *Acaulospora scrobiculata* (22.45%) under HAFP followed by *Claroideoglomus claroideum* (9.38%) both in HAFP and WlAFP type. *Acaulospora scrobiculata* (43.04%), *Claroideoglomus claroideum* (22.30%), *Glomus* spp.2 and *Acaulospora spinosa* (20.33%) were dominant in HAFP and TSWAFP, respectively. *Acaulospora scrobiculata* had higher IF, RA, and IV in HAFP (Table SI 1). The abundance of AMF species was higher in the upper soil depth of the HAFP **(**Table 3**)**. Moreover, the dominance of the forty-three morphospecies were shown by Whittaker plots (Fig. 2), and the relationship of AMF morphospecies composition and soil properties among AFP was illustrated using principal component analysis (Fig. 3).

**Table 3:** Isolation frequency (IF), Relative abundance (RA), importance value (IV) of arbuscular mycorrhizal fungi species among the four agroforestry practices between the upper (0–30 cm) and lower soil depths (30–60 cm)

Agroforestry types	IF		RA		IV	
	0–30	30–60	0–30	30–60	0–30	30–60
HAFP	24.728	0	5.638	0.08	15.482	0
CLAFP	12.369	1.563	2.958	0.284	7.665	0.924
WLAFP	12.271	0.596	2.547	0.117	7.411	0.383
TSWAFP	14.627	0	1.4944	0.109	9.516	0

*The AMF species were from the soil of homegarden based agroforestry practices (HAFP), cropland-based agroforestry practices (CLAFP), woodlot-based agroforestry practices (WlAFP) and trees on soil and water conservation-based agroforestry practices (TSWAFP) in southern Ethiopia

### AMF spore density, richness and diversity

The spore abundance was significantly different between AFP and soil depth (Table 4). The average number of AMF spore abundance was from 11.550 to 76.415 100 g dry soil^− 1^ in HAFP, 6.836 to 44.279 in ClAFP, 8.805 to 51.937 in WlAFP and 8.672 to 32.046 in TSWAFP (Table SI2). The maximum number of spore abundance was found in HAFP followed by WlAFP, ClAFP and TSWAFP from the upper soil depth (Table 4). In both upper and subsoil depths, the highest and lowest spore abundance was found associated with *Ficus vasta* trees in the HAFP and *Musa* species in the CLAFP, respectively. TSWAFP and CLAFP had a smaller number of AMF spores per 100 g of dry soil. The AFP-soil depth interaction effect was not significantly different (F = 0.34, P = 0.81). The number of AMF morphospecies observed under different AFP types ranged from 6 to 18 (Table 2). The HAFP had the highest number of morphospecies (18) followed by the WlAFP, TSWAFP, and ClAFP. The Shannon diversity index ranged from 1.35 to 1.86. WlAFP had the highest diversity followed by HAFP and the lowest diversity was recorded from ClAFP.

**Table 4:** The summary of mean spore abundance (mean ± SE) of arbuscular mycorrhizal fungi 100 g^− 1^ dry soil in each soil depth and AMF colonization under agroforestry-based practices (AFP) in the dry lands of southern Ethiopia

AFP	AMF spore density (100 g^− 1^ dry soil)		AMF root colonization (%)		
	0–30 cm	30–60 cm	AC	VC	HC
HAFP	3403.169^a^	2658.723^d^	12.719^a^	16.204^d^	24.485^e^
CLAFP	1936.367^b^	1754.880^b^	9.698^b^	12.144^a^	20.248^e^
WLAFP	2716.713^c^	2280.155^c^	15.282^c^	17.347^d^	28.244^f^
TSWAFP	1812.488^b^	1443.050^b^	7.147^b^	9.003^b^	15.516^c^

*Units within a column followed by the same superscript/s are not significantly different at *p* > 0.05. The AMF species were from the soil of homegarden based agroforestry practices (HAFP), cropland-based agroforestry practices (CLAFP), woodlot-based agroforestry practices (WlAFP) and trees on soil and water conservation-based agroforestry practices (TSWAFP) in drylands of southern Ethiopia

### AMF root colonization

All woody species sampled from HAFP, CLAFP, WLAFP, and TSWAFP were colonized by AMF. The percentages of AMF root colonization were significantly different between tree species (p < 0.05). The highest colonization was found in *Ficus vasta* (54.750%) under WlAFP, and the lowest colonization was recorded from the roots of *Vernonia auriculifera* woody species (2.075%) under TSWAFP (Table SI3).

### Soil physicochemical properties

The soil in the study area was slightly acidic, with mean pH values ranged from 6.20 in CLAFP to 7.07 in HAFP. The highest organic carbon content was found in HAFP followed by ClAFP. The lowest organic carbon was recorded in CLAFP. Soil total nitrogen concentration varied between 0.20 and 0.35%. pH, carbon, nitrogen, and silt concentrations were significantly different between the four AFP (Table 5).

**Table 5:** Soil Properties (mean + SE) under the four different agroforestry practices in drylands of Southern Ethiopia

		Type of Agroforestry Practices			
Soil Parameters	Depth (cm)	HAFP	ClAFP	WlAFP	TSWAFP
pH (1:2.5 soil: water)	0–30	7.07^a^	6.20^c^	6.34^bc^	6.39^abc^
	30–60	7.02^ab^	6.42^abc^	6.50^abc^	6.25^c^
Organic carbon (%)	0–30	3.62^a^	2.77^ab^	3.16^ab^	2.69^b^
	30–60	3.23^ab^	2.46^b^	2.87^ab^	2.77^ab^
Total nitrogen (%)	0–30	0.46^a^	0.23 ^ab^	0.31^ab^	0.20^ab^
	30–60	0.29 ^ab^	0.19^ab^	0.24^b^	0.22^ab^
Available phosphorus (ppm)	0–30	12.63^a^	13.84^a^	11.65^a^	12.07^a^
	30–60	11.06^a^	12.79^a^	10.66^a^	11.91^a^
Sand (%)	0–30	73.25^a^	67.25^a^	67.50^a^	65.25^a^
	30–60	69.88^a^	66.75^a^	67.63^a^	68.81^a^
Clay (%)	0–30	9.31^a^	10.00^a^	8.75^a^	8.00^a^
	30–60	8.69^a^	9.75^a^	9.31^a^	8.19^a^
Silt (%)	0–30	17.44^b^	22.75^ab^	23.75^ab^	26.75^a^
	30–60	21.44^ab^	23.50^ab^	23.69^ab^	23.00^ab^

*Units within a row followed by the same superscript/s are not significantly different at p > 0.05. The AMF species were from the soil of homegarden based agroforestry practices (HAFP), cropland-based agroforestry practices (CLAFP), woodlot-based agroforestry practices (WlAFP) and trees on soil and water conservation-based agroforestry practices (TSWAFP) in southern Ethiopia

### Relationship between AMF Spore composition, root colonization and soil physicochemical properties

The distribution of AMF spore composition and diversity were significantly related to soil organic carbon and total nitrogen. The soil organic carbon in the upper soil depth was significantly related to spore abundance of surface soil (β = 1646.889, P-value = 0.001, r^2^ = 0.312) and AMF diversity (β = 0.209, p-value = 0.023, r^2^ = 0.162) respectively. The total soil nitrogen had significant relationship with the spore abundance of surface soil (β = 9354.988, P-value = 0.038, r^2^ = 0.135) and AMF diversity (β = 2.839, P-value = 0.000, r^2^ = 0.403) respectively. The subsurface soil total nitrogen was significantly related with soil AMF diversity (β = 3.566, P-value = 0.000, r^2^ = 0.433). The distribution of AMF spores abundance and diversity were positively affected by both soil organic carbon and total nitrogen.

### Similarity in AMF composition

The similarity of soil AMF species under different AFP ranged from 0.385 to 0.556. The highest similarity in soil AMF occurred between TSWAFP and ClAFP (55.6%), followed by TSWAFP and WLAFP (50.0%). TSWAFP and ClAFP shared the highest number of morphospecies (55.6%), while the WlAFP and HAFP had the lowest (38.5%) similarity values of AMF species.

## Discussion

### AMF communities

The AMF species and morphotypes identified are common in tropical ecosystems. *Glomus*, *Funneliformis*, and *Claroideoglomus* genus were dominant in countries with tropical agroecology including in Ethiopia [72, 73], Cameroon [74], Kenya [75], and Sudan [76]. Similarly, *Acaulospora* and *Glomus* were dominant in tropical soils [33, 35, 72, 77, 78]. Comparable results were reported to the current findings with 42 AMF species belonging to 15 genera in Ethiopia [35], 42 AMF species belonging to 12 genera in Sudan [76] under different cropping systems, 43 species of AMF were isolated from the Western Brazilian Amazon [33], 41 AMF species and 5 morphotypes in Ethiopia [78]. The difference in AMF species/families between different areas and land use types could be due to the preference of AMF species to various host plants, abiotic factors and agroecological variation.

The AMF species diversity observed in this study was higher than the species identified from different land use types in the tropics: 21 AMF species in Brazil [79]; 9 AMF genera and 16 species in tropical savanna landscape of Tanzania [80]; 14 AMF species in tropical savanna in Kenya [73], 17 AMF species isolated from tropical humid highlands of Kenya [70], and 18 species in Kenya [81]. This may be due to the diversity and the type of coexisting plant species sampled and preferred by AMF species [82].

The number of AMF morphospecies found in HAFP and WlAFP was significantly higher than the number of morphospecies collected from CLAFP and STWAFP. HAFP and WlAFP are less disturbed and had high surface biodiversity. The hypothesis that predicted a positive effect of AFP on AMF spore abundance, root colonization and diversity is confirmed. This is consistent with the findings of Sorensen *et al.,.* [83] who reported that microbial diversity are greater in the AFS due to the ameliorative effects of trees and organic matter inputs. Mixtures of plant species in AF usually allow a larger diversity and/or abundance of mycorrhizal fungi than annual crop based systems [39]. Perennial plant species have maintained higher AMF spore diversity than annual crop based systems [84]. Agroforestry practices are better to support the abundant and diverse AMF community than conventionally managed agricultural systems [40, 41, 43]. Small number of morphospecies have been observed in annual crop based agricultural practices (CLAFP and STWFP) in which intensive tillage and application of fertilizers is common (Table 1). AMF species diversity was low in high input than low input mono-cropped fields [35]. Besides, compared to no-tillage, intensive plowing of the soil under the conventional cultivation system can negatively affect the AMF community [85]. This indicated that land use types with high intensity could change the nature of soil and may decrease AMF species richness and diversity [86].

### Occurrence, abundance and dominance of AMF morphospecies

*Acaulospora*, *Claroideoglomus*, *Glomus*, and *Funneliformis*, were the dominant genera, of which *Acaulospora scrobiculata* was often found in all AFP. *A scrobiculata* could adapt to a wide range of soils and host species [87]. *Funneliformis, Claroideoglomus*, and *Glomus* were the dominant genera that were reported [35, 72]. Moreover, *Acaulospora* and *Glomus* are dominant in dry tropical systems [33, 35, 72, 78]. The two AMF genera can adapt to wide environmental variables [88]. *Acaulospora scrobiculata* was distributed in all AFP types and categorized as generalists, while most of the AMF species that were found in this study were described as rare species. *Acaulospora scrobiculata* have the capability to sustain various biotic and abiotic changes.

### AMF spore abundance, richness and diversity

AMF spore abundance recorded in this study was comparable with the findings from different tropical areas [35, 42, 44, 78, 86]. The spore densities reported in this study were higher than findings from similar agroecologies [39, 89] and lower than those obtained by [90] in Ivory Coast. The differences might come from the variation in the level of soil disturbance [20].

In this study, the highest spore abundance was found under the HAFP type followed by the WlAFP as compared to annual crop-based AFP. Lower spore abundance was found in CLAFP and TSWAFP. The finding was consistent with previous research [75] who reported that an undisturbed areas harbor higher spore density in comparison to cropland based agricultural systems. Soil disturbance can reduce AM fungi spore densities [39, 48]. Land management, agroecological variation, AMF species type and host plants preferences might cause difference in AMF spore densities.

Substantial impacts of land management practices, variability in vegetation types and soil properties may bring variation in AMF diversity. The diversity of AMF species could be resulted from the heterogeneity among the habitats evaluated, land management practices, and the cropping systems [79, 85]. Systems that are less disturbed have higher AMF community diversity compared to monocrop based systems [91, 92]. Lower diversities were recorded in both CLAFP and STWAFP that might be due to soil disturbance caused by cultivation and the use of agricultural inputs that negatively affected AMF diversity. Land use intensity has negative impact on AMF diversity [74, 77, 93]. Intensifying land use by monocropping and increasing the fertilization practices can have a detrimental effect on AMF species diversity [94]. On the contrary, the conversion of the forest into other land uses in tropical Amazon did not reduce AM fungal diversity [33]. Similarly, tractor tillage has increased AMF diversity while zero tillage decreased diversity [95]. Some studies indicated that high intensity of land use did not change or even increased AMF diversity or species richness [75].

HAFP had the high AMF richness, while ClAFP and TSWAFP had low AMF richness. Higher richness of AMF were recorded from less disturbed land use systems [73]. Difference in land management conditions may cause differences in AMF spores density between different land use types [91]. *Acaulospora* and *Glomus* spores were dominant [39, 96] due to their broad distribution in agroecosystems [26]. AMF species richness recorded in this study was comparable with reports from different land use types in Ethiopia [38], and 43 species recorded in Brazil [33]. The species richness reported in this paper was higher compared to the 29 species identified from Southern Ethiopia [72], 9 species from Tanzania [809], and 12 species from Kenya [75]. AMF species richness could be determined by plant diversity, density and soil physicochemical properties [44].

### AMF root colonization

All sampled woody species were colonized by AMF structures which is consistent with reports from different land use types in the tropics [73] and low compared with the findings that reported 4 to 95%, [72, 75] and 3.5 to 96.3% root colonization [89]. Age of plant species, phenology of plant species, genetic variation among plant species and abiotic factors may contribute to the variation in AMF colonization [97]. Hyphal colonization was higher in all AFP followed by vesicular colonization while arbuscular colonization was low (Table SI3),which agrees with the findings of [78], and [47]. Hyphae are the primary structures of AMF and can exist for extended period. The AFP type with higher plant density and less disturbed (WLAFP and HAFP) increased root colonization, while low AMF root colonization was recorded under cropland-based AFP (CLAFP and TSWAFP). WLAFP and HAFP had high organic carbon and lower disturbance. Disturbance of the soil can decrease AMF root colonization. Soil disturbance and vegetation removal generally had the greatest impact on biological properties, including AMF root colonization [26]. Other studies reported no relationship between the degree of land use intensification and root colonization of AM fungi [98].

### The relationship between AMF composition and soil physicochemical properties

Soil properties have a considerable influence on AMF [26]. AMF spore abundance and diversity had a significant correlation with soil organic carbon and total nitrogen. This finding is consistent with [99] who reported that AMF spore abundance and distribution was mainly explained by TN and OC [100]. AMF abundance in soils increased with increasing soil macronutrient levels [101]. AMF community diversity and spore abundance were positively related with soil nitrogen [102, 103] and the soil organic carbon [104]. Additionally, the ecological processes indicated that the presence of strong relationship among the soil AMF community structures and OC [105]. This shows that OC and TN were good predictors of changes in AMF community in the study area.

### Similarity in AMF composition

The highest dissimilarity was found between AMF communities in HAFP and WlAFP (61.5%), while AMF communities in STWAFP and CLAFP had high similarity (55.6%). The higher similarity index of species composition between STWAFP and CLAFP is due to lower woody species diversity in the two practices. Cultivated land has high AMF species homogeneity [75] and disturbed ecosystem has lower biodiversity [106]. Similarly, there is a low diversity of the phylum Glomeromycota in disturbed and eroded areas [107]. Environmental conditions seemed to be more influential in determining the similarity of AMF communities than the abundance and diversity of vegetation in the area [108].

## Conclusion

In this study, the HAFP and WlAFP were the least disturbed AFPs and had the highest AMF spore density, root colonization and species diversity. The ClAFP and TSWAFP were the most disturbed and cultivated AFP that had the low woody species density with the low AMF spore density, root colonization and AMF species diversity. The AMF SD, RC and species diversity were significantly different among the different AFPs, due to both the plant host specificity of AMF and the difference in land management practices between the AFP. The difference in management intensity among AFP may result significant variation in AMF SD, RC, species diversity, species composition, dominance, and soil nutrients. The HAFP followed by WlAFP is an alternative AFP for in-situ surface and subsurface biodiversity resource conservation.

### Electronic supplementary material

Below is the link to the electronic supplementary material.

Supplementary Material 1

## Data Availability

The datasets generated during and/or analyzed during the current study are available from the corresponding author on reasonable request.
