# Supplementary material for: Diversity of Arbuscular Mycorrhizal fungi under different agroforestry practices in the drylands of Southern Ethiopia
Source: BMC Plant Biol. 2023 Dec 9;23:634. doi: 10.1186/s12870-023-04645-6 (PMC10709898; doi:10.1186/s12870-023-04645-6)
Supplement: Supplementary file 1 — Supplementary Material 1 [file 12870_2023_4645_MOESM1_ESM.docx]

**Supplementary information (SI)**

**Richness of Arbuscular Mycorrhizal Fungi under Different Agroforestry Practices in the dry lands of Southern Ethiopia**

# Nebiyou Masebo^1, 9^, Emiru Birhane^2, 8*^, Serekebirhan Takele^3^, Zerihun Belay^4^, Juan J.Lucena^5,^ Araceli Pérez Sanz^6^ and Agena Anjulo^7^

^1^Department of Natural Resource Management, Wolaita Sodo University, Ethiopia, P.O. Box 128, Wolaita Sodo , Ethiopia, Email: [nebiyou.masebo@amu.edu.et](mailto:nebiyou.masebo@amu.edu.et)

^2^ Department of Land Resource Management and Environmental Protection, Mekelle University, P.O. Box 231, Tigray, Ethiopia, Email: [emiru.birhane@mu.edu.et](mailto:emiru.birhane@mu.edu.et)

^3^ Department of Biology, Arba Minch University, Ethiopia, P.O. Box 138, Arba Minch,Ethiopia,Email: [sereke100@yahoo.com](mailto:sereke100@yahoo.com)

^4^Department of Applied Biology, Adama Science and Technology University, Ethiopia, P.O. Box 231, Adama, Ethiopia, Email: [zebelay2009@gmail.com](mailto:zebelay2009@gmail.com)

^5^ Department of Agricultural Chemistry and Food Science, Autonoma University of Madrid, Spain, Postal code: 28049 Madrid, Spain, Email: [juanjose.lucena@uam.es](mailto:juanjose.lucena@uam.es)

# 6 Department of Agricultural Chemistry and Food Science, Autonoma University of Madrid, Spain, Postal code: 28049 Madrid, Spain, Email: araceli.perezs@uam.es

^7^ Environment and Forest Research Institute, Addis Ababa, Ethiopia, P.O. Box 231, Addis Ababa, Ethiopia, Email: [agenaanj@yahoo.com](mailto:agenaanj@yahoo.com)

^8^ Institute of Climate and Society, Mekelle University, P.O.Box 231, Mekelle, Ethiopia: Email: [emiru.birhane@mu.edu.et](mailto:emiru.birhane@mu.edu.et)

^9^ Department of Biology, Arba Minch University, Ethiopia, P.O. Box 138, Arba Minch,Ethiopia,Email: [nebiyou.masebo@amu.edu.et](mailto:nebiyou.masebo@amu.edu.et)

# *Corresponding author: Emiru Birhane, Email: [emiru.birhane@mu.edu.et](mailto:emiru.birhane@mu.edu.et)

### Table SI 1: Distribution of Arbuscular mycorrhizal fungi Species among Different Agroforestry Practices in dry lands of Southern Ethiopia

| **AMF** | **IF** | | | | | | | | **RA** | | | | | | | | **IV** | | | | | | | |
| --- | --- | --- | --- | --- | --- | --- | --- | --- | --- | --- | --- | --- | --- | --- | --- | --- | --- | --- | --- | --- | --- | --- | --- | --- |
|  | **HAFP** | | **ClAFP** | | **WlAFP** | | **TSWAFP** | | **HAFP** | | **ClAFP** | | **WlAFP** | | **TSWAFP** | | **HAFP** | | **ClAFP** | | **WlAFP** | | **TSWA FP** | |
|  | D1 | D2 | D1 | D2 | D1 | D2 | D1 | D2 | D1 | D2 | D1 | D2 | D1 | D2 | D1 | D2 | D1 | D2 | D1 | D2 | D1 | D2 | D1 | D2 |
| *Acaulospora bireticulata*, F.M. Rothwell & Trappe) | 0 | 0 | 18.35 | 0 | 20.25 | 0 | 0 | 0 | 0 | 0 | 4.26 | 0 | 4.17 | 0 | 0 | 0 | 0 | 0 | 11.31 | 0 | 12.21 | 0 | 0 | 0 |
| *Acaulospora cavarnata* (Blaszk) | 20.25 | 0 | 0 | 0 | 0 | 0 | 0 | 0 | 4.17 | 0 | 0 | 0 | 0 | 0 | 0 | 0 | 12.21 | 0 | 0 | 0 | 0 | 0 | 0 | 0 |
| *Acaulospora denticulate* (Sieverd. & S. Toro) | 27.67 | 0 | 4.17 | 0 | 0 | 0 | 0 | 0 | 6.29 | 0 | 0.87 | 0 | 0 | 0 | 0 | 0 | 16.98 | 0 | 2.52 | 0 | 0 | 0 | 0 | 0 |
| *Acaulospora koskei* (B?aszk.) | 12.56 | 0 | 0 | 0 | 8.33 | 0 | 4.72 | 0 | 2.13 | 0 | 0 | 0 | 1.40 | 0 | 0.92 | 0 | 7.35 | 0 | 0 | 0 | 4.87 |  | 2.82 | 0 |
| *Acaulospora rehmii* (Sieverd. & S. Toro) | 0 | 0 | 0 | 0 | 0 | 4.17 | 0 | 0 | 0 | 0 | 0 | 0 | 0 | 0.82 | 0 | 0 | 0 | 0 | 0 | 0 | 0 | 2.50 | 0 | 0 |
| *Acaulospora scrobiculata* (Trappe) | 63.64 | 0 | 8.33 | 0 | 12.52 | 0 | 8.33 | 0 | 15.35 | 0 | 1.40 | 0 | 2.13 | 0 | 1.4 | 0 | 43.05 | 0 | 4.87 | 0 | 7.33 | 0 | 4.87 | 0 |
| *Acaulospora spinosa* (C. Walker & Trappe) | 32.50 | 0 | 0 | 0 | 25.56 | 0 | 0 | 0 | 8.17 | 0 | 0 | 0 | 0 | 0 | 0 | 0 | 20.34 | 0 | 0 | 0 | 0 | 0 | 0 | 0 |
| *Ambispora appendicula* (C. Walker) | 0 | 0 | 0 | 0 | 0 | 0 | 13.27 | 0 | 0 | 0 | 0 | 0 | 0 | 0 | 0 | 0 | 0 | 0 | 0 | 0 | 0 | 0 | 13.54 | 0 |
| *Ambispora leptoticha* (C. Walker, Vestberg & Schuessler) | 0 | 0 | 0 | 8.33 | 0 | 0 | 13.27 | 0 | 0 | 0 | 0 | 1.4 | 0 | 0 | 1.23 | 0.98 | 0 | 0 | 0 | 4.87 | 19.23 | 0 | 10.79 | 0 |
| *Claroideoglomus claroideum* (C. Walker & Schuessler) | 35.23 | 0 | 0 | 0 | 30.25 | 0 | 12.45 | 0 | 9.38 | 0 | 0 | 0 | 8.21 | 0 | 1.69 | 0 | 22.31 | 0 | 0 | 0 | 0 | 0 | 7.07 | 0 |
| *Claroideoglomus etunicatum* (C. Walker & Schuessler) | 25.42 | 0 | 30.35 | 0 | 0 | 0 | 0 | 0 | 5.33 | 0 | 9.25 | 0 | 0 | 0 | 0 | 0 | 15.38 | 0 | 19.80 | 0 | 0 | 0 | 0 | 0 |
| *Claroideoglomus luteum* (C. Walker & Schuessler | 0 | 0 | 0 | 0 | 4.17 | 0 | 8.33 | 0 | 0 | 0 | 0 | 0 | 0.82 | 0 | 1.13 | 0 | 0 | 0 | 0 | 0 | 2.50 | 0 | 7.37 | 0 |
| *Funneliformis geosporum* (C. Walker & Schuessler | 0 | 0 | 0 | 0 | 8.33 | 0 | 0 | 0 | 0 | 0 | 0 | 0 | 1.40 | 0 | 0 | 0 | 0 | 0 | 0 | 0 | 4.87 | 0 | 0 | 0 |
| *Funneliformis mosseae* (C. Walker & Schuessler | 32.63 | 0 | 0 | 0 | 4.65 | 0 | 30.67 | 0 | 8.15 | 0.96 | 0 | 0 | 0 | 0 | 3.95 | 0 | 20.39 | 0 | 0 | 0 | 2.81 | 0 | 17.31 | 0 |
| *Gigaspora gigantea* (Gerd. & Trappe) | 0 | 0 | 15.25 | 0 | 8.33 | 0 | 0 | 0 | 0 | 0 | 3.21 | 0 | 1.40 | 0 | 0 | 0 | 0 | 0 | 0 | 0 | 4.87 | 0 | 0 | 0 |
| *Glomus* spp 1 | 14.65 | 0 | 0 | 0 | 12.52 | 0 | 24.35 | 0 | 2.87 | 0 | 0 | 0 | 2.13 | 0 | 1.5 | 0 | 8.76 | 0 | 0 | 0 | 7.33 | 0 | 12.93 | 0 |
| *Glomus* spp 2 | 10.25 | 0 | 0 | 0 | 0 | 0 | 16.25 | 0 | 1.7 | 0 | 0 | 0 | 0 | 0 | 1.63 | 0 | 5.98 | 0 | 0 | 0 | 0 | 0 | 8.94 | 0 |
| *Paraglomus occultum* (J.B. Morton & D. Redecke) | 0 | 0 | 22.50 | 0 | 0 | 0 | 0 | 0 | 0 | 0 | 4.67 | 0 | 0 | 0 | 0 | 0 | 0 | 0 | 0 | 0 | 13.59 | 0 | 0 | 0 |
| *Rhizophagus aggregatus* (C. Walker | 17.28 | 0 | 0 | 0 | 21.35 | 0 | 0 | 0 | 3.15 | 0 | 0 | 0 | 4.36 | 0 | 0 | 0 | 10.22 | 0 | 9.23 | 0 | 12.86 | 0 | 0 | 0 |
| *Scutellospora* spp | 0 | 0 | 0 | 4.17 | 0 | 0 | 0 | 0 | 0 | 0 | 0 | 0.87 | 0 | 0 | 0 | 0 | 0 | 0 | 0 | 2.52 | 0 | 0 | 0 | 0 |
| *Septoglomus constrictum* (Sieverd., G. A. Silva & Oehl) | 4.65 | 0 | 0 | 0 | 15.67 | 0 | 0 | 0 | 0.96 | 0 | 0 | 0 | 3.35 | 0 | 0 | 0 | 2.81 | 0 | 0 | 0 | 9.51 | 0 | 0 | 0 |
| Unidentified species | 0 | 0 | 0 | 0 | 0 | 4.17 | 0 | 0 | 0 | 0 | 0 | 0 | 0 | 0.82 | 0 | 0 | 0 | 0 | 0 | 0 | 0 | 2.50 | 0 | 0 |

*****The AMF species were from the soil of homegarden based agroforestry practices (HAFP), cropland based agroforestry practices (CLAFP), woodlot based agroforestry practices (WlAFP) and trees on soil and water conservation based agroforestry practices (TSWAFP) in southern Ethiopia. Isolation frequency (IF) was calculated as the number of samples in which the given species was isolated as the percentage of the total number of samples. Relative abundance (RA) was calculated as the number spores of a given AMF species as a percentage of the total number of spores and the important value was calculated as IV= (IF + RA)/2. D1=upper depth 0-30 cm), & D2=lower depth (30-60 cm).


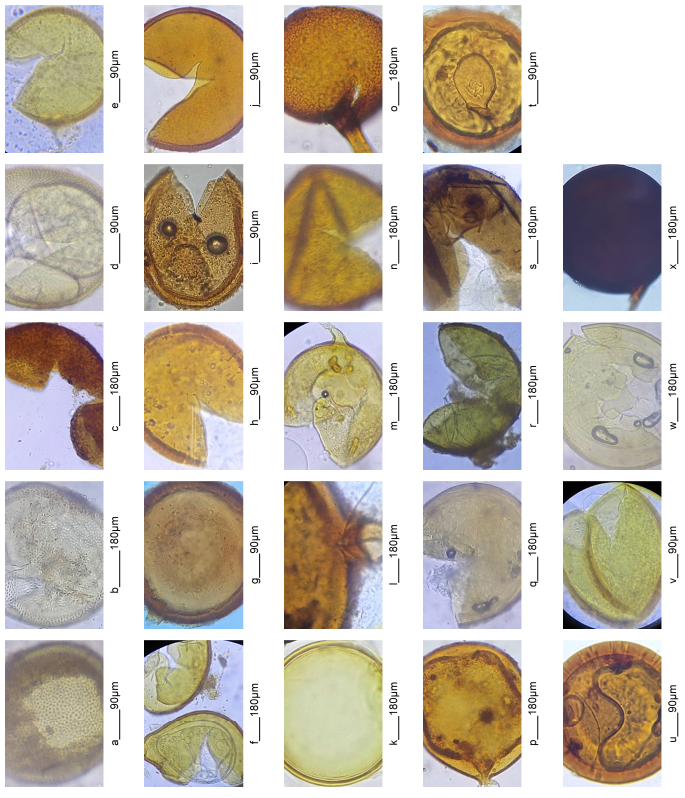


**Figure SI 1:** Microscopic photos of some AMF morphotypes **(**a=*Acaulospora cavernata*,b=*Acaulospora cavernata*,c=Acaulospora rehmii,d=*Acaulospora scrobiculata,* e=*Acaulospora spinosa*, f=*Acaulospora spinosa*,g=*Calroideoglomus* *calroideum*,h=*Calroideoglomus etunicatum,* i=*Caroideoglomus ethunicatum*,j=*Caroideoglomus* *ethunicatum*, k=*Cetraspora pellucida*,l=*Dentiscutata rubra*, m=*Funneliform mosseae*, n=*Funneliformis geosporum*, o=*Funnelisformis caledonius*, p=*Funnelisformis mosseae*, q=*Gigaspora albida*, r=*Gigaspora gigantean*,s=*Gigaspora rosea*, t=*Glomus mosseae, u=Glomus* spp 2, v=*Rhizophagus aggregatus*.w=*Pasospora* *pellucida* ,x=*Scutulo spora).*

**able SI 2**: The mean spore density (mean + SE) of Arbuscular mycorrhizal fungi 100 g^-1^ dry soil in each soil depth between different woody species under Agroforestry based practices in dry lands of southern Ethiopia

| Type of Agroforestry Based Practices | | | | | |
| --- | --- | --- | --- | --- | --- |
| **HAFP** | | | **ClAFP** | | |
| **Plant Species** | **Soil Depth (cm)** | | **Plant Species** | **Soil Depth (cm)** | |
|  | **0-30** | **30-60** |  | **0-30** | **30-60** |
| *Ficus vasta* Forssk*.* | 7641.5**^a^** (+255.1) | 5845.5**^bc^** (+394.1) | *Ficus vasta* Forssk. | 4427.9^a^ (+0.576) | 3839.8^b^ (+1.029) |
| *Cordia africana* Lam*.* | 6219.0**^b^** (+206.1) | 5112.2**^cde^** (+235.4) | *Cajanus cajan (L.)* Mill | 2718.5^c^ (+0.652) | 2273.9^de^ (+0.704) |
| *Croton macrostachyus* Del. | 6171.7**^b^** (+471.0) | 4506.3**^def^** (+247.3) | *Acacia abyssinica* Hochst | 2761.0^cd^ (+1.297) | 2313.5^cde^ (+0.767) |
| *Erythrina brucei* Schweinf | 5346.8**^bcd^** (+233.8) | 4164.3**^ef^** (+336.7) | *Cordia africana* Lam*.* | 2741.4^cd^ (+0.287) | 2157.0^ef^ (+0.628) |
| *Grevillea robusta* R. Br*.* | 3628.5**^fg^** (+218.2) | 2997.0**^gh^** (+166.8) | *Sassbania sesbaniya* | 2364.1^cde^ (+0.812) | 1599.8^gh^ (+0.603) |
| *Ricinus communis* L. | 2544.5**^hi^** (+248.3) | 1554.5**^ijk^** (+41.3) | *Erythrina brucei* Schweinf | 2313.8^cde^ (+0.936) | 1550.0^gh^ (+0.676) |
| *Coffea arabica* L. | 2266.3**^hij^** (+118.4) | 1811.1**^ijk^** (+141.2) | *Croton macrostachyus* Del. | 1816.8^fg^ (+0.696) | 1129.2^hijk^ (+0.689) |
| *Millettia ferruginea* Hochst | 2037.5**^hijk^** (+240.1) | 1603.0**^ijk^** (+193.6) | *Coffea arabica* L. | 1526.6^gh^ (+0.143) | 1254.7^ghi^ (+1.282) |
| *Persea americana* Mill. | 1945.5**^ijk^** (+295.8) | 1716.3^i^**^jk^** (+284.8) | *Grevillea robusta* R. Br. | 1267.6^hij^ (+1.022) | 637.1^l^ (+0.042) |
| *Musa* | 1649.8^i^**^jk^** (+209.8) | 1292.0**^jk^** (+206.2) | *Persea americana* Mill. | 1171.2^hijk^ (+1.273) | 747.3^jkl^ (+1.033) |
| *Mangifera indica* L*.* | 1636.3**^ijk^** (+99.3) | 1324.7**^jk^** (+59.3) | *Mangifera indica* L. | 977.3^ijkl^ (+2.455) | 625.5^l^ (+0.575) |
| *Vernonia auriculifera* Hier*n* | 1564.5**^ijk^** (+245.8) | 1481.5**^jk^** (+193.3) | *Vernonia auriculifera* Hiern | 964.5^ijkl^ (+2.323) | 541.9^l^ (+0.281) |
| *Ensete ventricosum(*Welw.*)* | 1589.3**^ijk^** (+46.0) | 1155.0**^k^** (+85.4) | *Musa* | 927.1^ijkl^ (+0.725) | 683.6^kl^ (+0.411) |

| ***Continued… (***The mean spore density mean + SE). | | | | | |
| --- | --- | --- | --- | --- | --- |
| **WlAFP** | | | **TSWAFP** | | |
| **Plant Species** | **Soil Depth (cm)** | | **Plant Species** | **Soil Depth (cm)** | |
|  |  | |  |  | |
|  |  |  |  |  | |
|  | **0-30** | **30-60** |  | **0-30** | **30-60** |
| *Ficus vasta* Forssk*.* | 5193.7^a^ (+1.1.596) | 4776.3^bc^ (+0.915) | *Cordia africana* Lam. | 3204.6^a^ (+1.464) | 2383.2^b^ (+1.150) |
| *Cordia africana* Lam*.* | 5091.2^ab^ (+1.926) | 4209.3^de^ (+1.663) | *Sassbania sesbaniya* | 2565.5^b^ (+0.8843) | 2153.9^bc^ (+0.703) |
| *Cajanus cajan (L.)* Mill | 4800.0^bc^ (+1.552) | 4499.3^cd^ (+1.269) | *Croton macrostachyus* Del. | 2363.4^bc^(+0.528) | 2213.4^cd^ (+0.980) |
| *Croton macrostachyus* Del*.* | 3656.2^ef^ (+1.569) | 2586.7^ijk^ (+0.639) | *Grevillea robusta* R. Br. | 1834.1^d^ (+0.945) | 1212.2^ef^ (+0.649) |
| *Acacia abyssinica* Hochst | 3410.6^fg^ (+1.643) | 2682.5^jkl^ (+1.208) | *Persea americana* Mill. | 1200.1^e^ (+0.421) | 1029.1^ef^ (+0.707) |
| *Coffea arabica* L*.* | 3312.4^fgh^ (+1.344) | 2204.2^kl^ (+0.928) | *Musa* | 1190.6^e^ (+0.6648) | 938.7^ef^ (+0.578) |
| *Eucalyptus camaldulensis* | 2971.0^ghi^ (+1.083) | 2892.0^hij^ (+1.433) | *Vernonia auriculifera* Hiern | 1140.7^ef^ (+2.282) | 746.7^f^ (+0.342) |
| *Grevillea robusta R*. Br*.* | 2596.8^ijk^ (+0.958) | 2093.2^kl^ (+1.086) | *Mangifera indica* L*.* | 1000.9^ef^ (+0.414) | 867.2^ef^ (+0.652) |
| *Eucalyptus globulus* | 24.24.375^jkl^(+0.912) | 2100.8^lm^ (+0.938) |  |  |  |
| *Juniperus procern* Hochst. ex Endl*.* | 2084.0^lm^ (+0.983) | 1581.5^no^ (+0.656) |  |  |  |
| *Erythrina brucei* Schweinf | 1544.3^mn^ (+0.381) | 1104.6^opq^ (+0.682) |  |  |  |
| *Mangifera indica* L*.* | 1487.5^no^ (+0.659) | 1253.675^nopq^ (+1.056) |  |  |  |
| *Cupressus lusitanica* Mill. | 1391.0^nop^ (+0.728) | 1232.1^opq^ (+0.821) |  |  |  |
| *Persea americana* Mill. | 1160.0^nopq^ (+0.997) | 1188.7^nopq^ (+0.518) |  |  |  |
| *Vernonia auriculifera* Hiern | 1035.3^opq^ (+0.477) | 1197.1^nopq^ (+3.125) |  |  |  |
| *Musa* | 1016.7^pq^ (+0.529) | 880.5^q^ (+0.879) |  |  |  |

*Units within a column followed by the same superscripts are not significantly different at *p*>0.05. The AMF species were from the soil of homegarden based agroforestry practices (HAFP), cropland based agroforestry practices (CLAFP), woodlot based agroforestry practices (WlAFP) and trees on soil and water conservation based agroforestry practices (TSWAFP) in dry lands of southern Ethiopia.

**Table SI 3:** The Arbuscular mycorrhizal fungi root colonization structures (%) between different woody tree species under different agroforestry practices in of southern Ethiopia.

| Plant species | The AMF root colonization structures (%) under **HAFP** | | | | Plant species | The AMF root colonization structures (%) under **ClAFP** | | | |
| --- | --- | --- | --- | --- | --- | --- | --- | --- | --- |
|  | AC | VC | HC | TRC |  | AC | VC | HC | TRC |
| *Ficus vasta* Forssk. | 21.075^a^(+0.075) | 19.8^d^ (+0.316) | 30.375^d^(+0.125) | 23.75^a^ (+3.33) | *Sassbania sesbaniya* | 16.075^a^(+0.217) | 14.375^d^(+0.315) | 37.475^a^(+0.368) | 22.64^a^(+7.43) |
| *Millettia ferruginea* Hochst | 20.300^a^(+0.834) | 31.900^a^(+1.502) | 35.400^b^(+0.804) | 29.20^b^(+4.56 | *Ficus vasta* Forssk. | 15.050^a^(+0.738) | 18.075^a^(+0.179) | 32.975^a^(+1.191) | 22.03^a^(+5.54) |
| *Ensete ventricosum* (Welw.) | 18.625^b^(+0.125) | 27.950^b^(+0.333) | 48.250^a^(+0.104) | 31.61^b^(+8.75) | *Cordia africana* Lam. | 12.125^b^(+1.053) | 18.300^a^(+1.054) | 21.475^e^(+1.529) | 17.3^b^(+2.75) |
| *Cordia africana* Lam. | 16.300^b^(+0.122) | 21.225^c^(+0.085) | 33.550^c^(+0.210) | 23.69^a^(+5.13) | *Erythrina brucei* Schweinf | 12.075^c^(+1.228) | 15.450^cd^(+0.738) | 21.750^f^(+1.410) | 16.43^b^(+2.84) |
| *Croton macrostachyus* Del. | 14.225^b^(+0.317) | 16.875^e^(+0.125) | 22.125^g^(+0.197) | 17.74^c^(+2.32) | *Cajanus cajan* (L.) Mill | 11.175^d^(+0.828) | 14.750^b^(+0.477) | 30.425^d^(+1.036) | 18.78^b^(+5.92) |
| Musa | 12.100^c^(+0.652) | 16.525^e^(+0.423) | 24.500^f^(+0.420) | 17.71^c^(+3.63) | *Grevillea robusta* R. Br. | 10.925^de^(+0.602) | 10.500^e^(+0.540) | 17.075^h^(+0.634) | 12.83^c^(+2.12) |
| *Erythrina brucei* Schweinf | 11.850^c^(+0.384) | 15.450^f^(+0.340) | 25.100^f^(+0.100) | 17.45^c^(+3.96) | *Acacia abyssinica* Hochst | 10.025^e^(+0.640) | 14.825^c^(+0.568) | 30.175^c^(+1.093) | 18.34^b^(+6.08) |
| *Coffea arabica* L. | 11.075^c^(+0.075) | 14.950^f^(+0.166) | 27.225^e^(+0.103) | 17.75^c^(+4.87) | *Croton* *macrostachyus* Del. | 10.075^e^(+0.249) | 15.400^c^(+0.286) | 15.225^i^(+0.131) | 13.57^c^(+1.75) |
| *Vernonia auriculifera* Hiern | 10.775^c^(+0.793) | 10.050^h^(+0.401) | 15.025^j^(+0.025) | 11.95^d^(+1.55) | *Persea americana* Mill. | 10.300^e^(+0.238) | 8.625^f^(+0.125) | 12.625^j^(+0.312) | 10.52^c^(+1.16) |
| *Grevillea robusta* R. Br. | 10.150^c^(+0.210) | 13.150^g^(+0.095) | 18.300^h^(+0.122) | 13.87^d^(+2.38 | *Coffea arabica* L. | 6.275^f^ (+0.304) | 11.575^e^(+1.011) | 19.800^g^(+0.937) | 9.63^c^(+3.93) |
| *Persea americana* Mill. | 9.575^c^(+0.388) | 12.025^g^(+0.668) | 16.725^i^(+0.602) | 12.78^d^(+2.10) | *Musa* | 6.000^f^(+0.29) | 7.800^f^(+0.108) | 11.050^k^(+0.411) | 8.28^c^(+1.48) |
| *Mangifera indica* L. | 6.525^d^(+0.184) | 5.850^i^(+0.287) | 13.100^k^(+0.402) | 8.49^e^(+2.31) | *Mangifera indica* L. | 3.225^g^(+0.209) | 3.575^h^(+0.125) | 7.200^l^(+0.091) | 4.67^d^(+1.27) |
| *Ricinus communis* L. | 2.775^d^(+0.304) | 4.900^i^(+0.274) | 8.625^l^(+0.409) | 5.43^e^(+1.71) | *Vernonia auriculifera* Hiern | 2.750^g^(+0.144) | 4.625^g^(+0.125) | 5.975^m^(+0.342) | 4.45^d^(+0.94) |

***Continued…* (**The AMF root colonization structures)**.**

| The AMF root colonization structures (%) under **WlAFP** | | | | | The AMF root colonization structures (%) under **TSWAFP** | | | | |
| --- | --- | --- | --- | --- | --- | --- | --- | --- | --- |
| Plant species | AC | VC | HC | TRC | Plant species | AC | VC | HC | TRC |
| *Cajanus cajan* (L.) Mill | 22.275^a^(+0.457) | 21.625d(+0.774) | 42.415^c^(+0.405) | 28.77^a^(+6.82) | *Sassbania sesbaniya* | 13.025^a^(+0.782) | 14.775^a^(+0.433) | 39.625^a^(+1.434) | 23.06^a^(+8.59) |
| *Coffea arabica* L. | 21.300^ab^(+0.122) | 25.325^ab^(+0.522) | 33.775^d^(+0.249) | 26.81^a^(+3.68) | *Cordia africana* Lam. | 11.550^b^(+0.556) | 15.925^a^(+0.229) | 19.225^b^(+1.219) | 15.57^b^(+2.22) |
| *Ficus vasta* Forssk. | 21.950^b^(+0.253) | 26.625^a^(+0.554) | 54.750^a^(+2.470) | 34.44^b^(+10.24) | *Croton* *macrostachyus* Del. | 9.975^c^(+0.643) | 7.175^c^(+0.871) | 14.125^c^(+0.239) | 10.43^c^(+2.02) |
| *Cordia africana* Lam. | 20.750^bc^(+0.250) | 23.075^c^(+0.075) | 35.810^d^(+0.274) | 26.55^a^(+4.68) | *Grevillea robusta* R. Br. | 6.150d(+0.747) | 9.675^b^(+0.568) | 10.600^d^(+0.726) | 8.81^c^(+1.36) |
| C*roton macrostachyus* Del. | 20.750^c^(+0.25) | 23.075^c^(+0.075) | 35.800^d^(+0.274) | 26.54^a^(+4.68) | *Persea americana* Mill. | 5.624^d^(+0.314) | 8.275^bc^(+0.319) | 14.425^c^(+0.433) | 9.44^c^(+2.61) |
| *Acacia abyssinica* Hochst | 18.700^d^(+0.332) | 23.125^c^(+0.125) | 46.825^b^(+0.609) | 29.55^a^(+8.73) | *Musa* | 5.575^d^(+0.405) | 8.325^bc^(+0.309) | 12.600^cd^(+0.743) | 8.83^c^(+2.04) |
| *Musa* | 15.250^e^(+0.722) | 21.775^d^(+0.578) | 31.850^d^(+1.056) | 22.96^c^(+4.83) | *Mangifera indica* L. | 3.200^e^(+0.283) | 4.375^d^(+0.315) | 8.700^e^(+0.441) | 5.43^d^(+1.67) |
| *Erythrina brucei* Schweinf | 15.200^e^(+0.356) | 21.200^d^(+0.122) | 34.600^d^(+0.204) | 23.67^c^(+5.73) | *Vernonia auriculifera* Hiern | 2.075^e^(+0.138) | 3.500^d^(+0.216) | 4.825^f^(+0.283) | 3.47^d^(+0.79) |
| *Persea americana* Mill. | 14.650^ef^(+0.830) | 12.750^f^(+0.661) | 21.850^e^(+0.366) | 16.35^d^(+2.77) |  |  |  |  |  |
| *Grevillea robusta* R. Br. | 14.458^ef^(+0.357) | 17.425e(+0.375) | 29.575^d^(+1.57) | 20.48^c^(+4.62) |  |  |  |  |  |
| *Vernonia auriculifera* Hiern | 14.225^f^(+0.578) | 13.125^f^(+0.554) | 20.725^e^(+0.796) | 16.03^d^(+2.37) |  |  |  |  |  |
| Eucalyptus ca*maldulensis* | 13.050^g^(+0.050) | 10.475^g^(+0.025) | 15.050^f^(+0.050) | 12.86^e^(+1.32) |  |  |  |  |  |
| *Eucalyptus globulus* | 11.550^gh^(+0.477) | 12.625^f^(+0.427) | 13.150^f^(+0.524) | 12.45^e^(+0.47) |  |  |  |  |  |
| *Mangifera indica* L. | 9.750^h^(+0.596) | 8.650^h^(+0.411) | 13.175^f^(+0.312) | 10.53^e^(+1.36) |  |  |  |  |  |
| *Juniperus procern* Hochst. ex Endl. | 5.575^i^ (+0.239) | 8.050^h^(+0.210) | 10.5^f^(+0.802) | 8.04^e^(+1.42) |  |  |  |  |  |
| *Cupressus lusitanica* Mill. | 5.075^i^(+0.275) | 8.625^h^(+0.197) | 12.050^f^(+0.050) | 8.58^e^(+2.01) |  |  |  |  |  |

*Units within a column followed by the same superscripts are not significantly different at *p>*0.05, total root colonization (TRC); The AMF root colonization structures were from the dominant woody species of homegarden based agroforestry practices (HAFP), cropland based agroforestry practices (CLAFP), woodlot based agroforestry practices (WlAFP) and trees on soil and water conservation based agroforestry practices (TSWAFP) in dry lands of southern Ethiopia
